# A Klebsiella pneumoniae Regulatory Mutant Has Reduced Capsule Expression but Retains Hypermucoviscosity

**DOI:** 10.1128/mBio.00089-19

**Published:** 2019-03-26

**Authors:** Kimberly A. Walker, Taryn A. Miner, Michelle Palacios, Dominika Trzilova, Daniel R. Frederick, Christopher A. Broberg, Victoria E. Sepúlveda, Joshua D. Quinn, Virginia L. Miller

**Affiliations:** a Department of Microbiology and Immunology, University of North Carolina School of Medicine, Chapel Hill, North Carolina, USA; b Department of Genetics, University of North Carolina School of Medicine, Chapel Hill, North Carolina, USA; Emory University School of Medicine

**Keywords:** HMV, RmpA, RmpC, capsular polysaccharide, hypervirulent

## Abstract

The polysaccharide capsule is an essential virulence factor for Klebsiella pneumoniae in both community-acquired hypervirulent strains as well as health care-associated classical strains that are posing significant challenges due to multidrug resistance. Capsule production is known to be transcriptionally regulated by a number of proteins, but very little is known about how these proteins collectively control capsule production. RmpA and RcsB are two known regulators of capsule gene expression, and RmpA is required for the hypermucoviscous (HMV) phenotype in hypervirulent K. pneumoniae strains. In this report, we confirmed that these regulators performed their anticipated functions in the ATCC 43816 derivative, KPPR1S: *rcsB* and *rmpA* mutants are HMV negative and have reduced capsule gene expression. We also identified a novel transcriptional regulator, RmpC, encoded by a gene near *rmpA*. The Δ*rmpC* strain has reduced capsule gene expression but retains the HMV phenotype. We further showed that a regulatory cascade exists in which KvrA and KvrB, the recently characterized MarR-like regulators, and RcsB contribute to capsule regulation through regulation of the *rmpA* promoter and through additional mechanisms. In a murine pneumonia model, the regulator mutants have a range of colonization defects, suggesting that they regulate virulence factors in addition to capsule. Further testing of the *rmpC* and *rmpA* mutants revealed that they have distinct and overlapping functions and provide evidence that HMV is not dependent on overproduction of capsule. This distinction will facilitate a better understanding of HMV and how it contributes to enhanced virulence of hypervirulent strains.

## Abstract (precis)

Klebsiella pneumoniae continues to be a substantial public health threat due to its ability to cause health care-associated and community-acquired infections combined with its ability to acquire antibiotic resistance. Novel therapeutics are needed to combat this pathogen, and a greater understanding of its virulence factors is required for the development of new drugs. A key virulence factor for K. pneumoniae is the capsule, and community-acquired hypervirulent strains produce a capsule that causes hypermucoidy. We report here a novel capsule regulator, RmpC, and provide evidence that capsule production and the hypermucoviscosity phenotype are distinct processes. Infection studies showing that this and other capsule regulator mutants have a range of phenotypes indicate that additional virulence factors are in their regulons. These results shed new light on the mechanisms controlling capsule production and introduce targets that may prove useful for the development of novel therapeutics for the treatment of this increasingly problematic pathogen.

## INTRODUCTION

Klebsiella pneumoniae is a pathogenic bacterium that commonly causes infections in a variety of sites, including the urinary tract, lungs, liver, and bloodstream (reviewed in reference 1). Although hypervirulent (*hv*) strains are known to cause community-acquired infections, K. pneumoniae is more commonly associated with nosocomial infections, as individuals in health care settings tend to be more susceptible to opportunistic pathogens. K. pneumoniae is also a member of the carbapenem-resistant Enterobacteriaceae (CRE), a group of Gram-negative pathogens that have acquired resistance to this important therapeutic drug. This problem of resistance prompted the CDC to classify CRE bacteria as an urgent threat; at the time of that report, K. pneumoniae was responsible for nearly 90% of CRE infections (3). K. pneumoniae isolates have also been found to be resistant to colistin, and they frequently produce extended-spectrum β-lactamases (ESBLs), making them resistant to most β-lactams currently in use (5).

Despite decades of research on K. pneumoniae, its repertoire of known virulence factors remains small compared to other pathogens. This organism generally possesses type 1 and type 3 fimbriae, lipopolysaccharide, siderophore iron acquisition systems, and a polysaccharide capsule that are necessary for virulence in animal models (1). More recently, K. pneumoniae isolates have been broadly classified as classical or *hv* (1, 4). The classical strains are commonly found circulating through health care facilities and tend to possess resistance to numerous antibiotics. *hv* strains are less commonly associated with antibiotic resistance, but they are considered community acquired as they can infect healthy hosts, often manifesting as pyogenic liver abscesses (reviewed in reference 4). Unlike *hv* strains, classical strains typically do not cause lethal disease in mouse models (11, 13). Two features that are known to distinguish classical from *hv* strains are the number of siderophore systems and the abundance of capsule (4). Classical strains typically have one or two siderophore systems, whereas *hv* strains have three or four, and *hv* strains are known to produce a very thick capsule associated with a hypermucoid phenotype called hypermucoviscosity (HMV). Although more than 130 capsule types have been identified (18), *hv* strains most frequently are type K1 or K2 (2; reviewed in references 1 and 4). Of particular concern is the recent emergence of strains that are both multidrug resistant and *hv* (6). The genes encoding resistance and genes associated with hypervirulence are often found on plasmids, suggesting that the frequency of strains carrying both virulence traits is likely to increase due to the ease with which these DNA elements are acquired.

The production of capsule appears to be a carefully orchestrated process. The genes encoding the proteins responsible for sugar synthesis, polymerization, and transport through the inner and outer membrane are transcriptionally regulated by several proteins. The Rcs phosphorelay system, first identified as a regulator of colonic acid synthesis in Escherichia coli (7), regulates capsule gene (*cps*) expression in a number of organisms, including *Klebsiella* (8, 9). Recent studies implicate H-NS (10), CRP (12), the iron-responsive regulator IcrR (14), and the response regulators KvgA, KvhA, and KvhR (16). RmpA was first reported in 1989 as a regulator of the mucoidy phenotype (17) and has since been linked to *hv* strains possessing the HMV phenotype, but its exact role is not understood (19). RmpA is encoded either on a virulence plasmid, the chromosome (on the ICEKp genomic island), or both (17, 20–22). We recently reported two new regulators of *cps* expression, KvrA and KvrB (23). The *kvrA* and *kvrB* genes are found in both classical and *hv* strains, but they appear to regulate capsule synthesis only in *hv* strains. This work sparked our interest in capsule regulation and in examining this process in more detail. From these endeavors, we identified RmpC as a novel protein that contributes to capsule regulation in KPPR1S, a derivative of the hypervirulent strain ATCC 43816. In addition, we confirmed the roles of RcsB and RmpA in capsule gene expression and the HMV phenotype in this strain. Through a series of epistasis experiments, we found that *rmpA* expression is dependent on RcsB, KvrA, and KvrB, but the roles of these proteins in capsule production are not limited to controlling *rmpA* expression. Furthermore, we found that loss of the *rmpC* gene results in decreased capsule gene expression, but curiously, it retains the HMV phenotype. This study provides evidence that capsule production is a distinct process from the process leading to the HMV phenotype. Understanding that these are separate processes will allow for specific probing of the HMV phenotype and how it contributes to enhanced virulence of *hv* strains.

## RESULTS

### *rmpA*, *rcsB*, and *rmpC* are required for normal capsule production.

**FIG 1:**
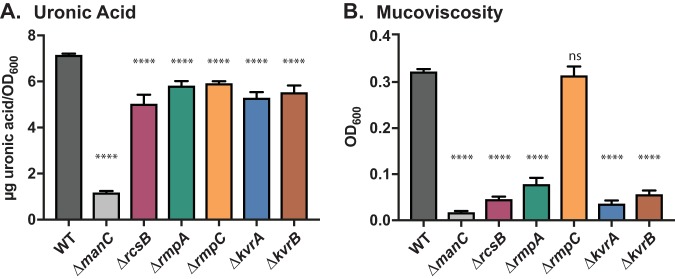
Capsule production and mucoviscosity are reduced in the DBD mutants. Saturated overnight cultures were subcultured into fresh M9-CAA and grown for 6 h at 37°C. Uronic acid (A) and mucoviscosity (B) were assessed as described in Materials and Methods. The data presented here are from a representative assay. The strains used are KPPR1S (wild type [WT]), VK506 (Δ*manC*), VK248 (Δ*rcsB*), VK352 (Δ*rmpA*), VK487 (Δ*rmpC*), VK277 (Δ*kvrA*), and VK410 (Δ*kvrB*). The one-way ANOVA test was performed to determine statistically significant differences between each mutant and WT. ****, *P ≤ *0.0001; ns, not significant.

Our identification of KvrA and KvrB as regulators of capsule (*cps*) gene expression (23) combined with the importance of capsule for virulence prompted us to explore *cps* regulation in more detail. Due to the heterogeneity of K. pneumoniae genomes, we examined the roles of the regulators RmpA and RcsB in *cps* expression in K. pneumoniae KPPR1S to determine whether their predicted roles were maintained in this strain. In closely examining the region around the *rmpA* gene (VK055_5097), we noted a neighboring gene with a predicted LuxR-type DNA binding domain (VK055_5099; designated *rmpC*). Its proximity to *rmpA* hinted at a related function, so it was targeted for deletion, and the effect of this mutation on capsule was examined along with strains carrying deletions of the *rmpA* and *rcsB* genes. Strains carrying gene deletions of the recently reported regulators KvrA and KvrB (23) were included for comparison. Because each of these genes encodes a putative DNA binding domain (DBD), they are collectively referred to as DBD mutants. These mutants were first assessed for HMV using the string test, where a colony is touched with a loop and lifted upward; colonies that stretch at least 5 mm are string test positive (24). Unlike the mutations in *rmpA* and *rcsB* (and *kvrA* and *kvrB*), the Δ*rmpC* strain was still mucoid and showed a positive string test result; like Δ*kvrA* and Δ*kvrB* mutant strains, Δ*rmpA* and Δ*rcsB* mutants were string test negative. As the string test is purely qualitative, we next performed two quantitative assays for capsule. Uronic acid (UA) is a key component of many capsules and has historically been used as an indicator of capsule content. UA was measured in KPPR1S and mutant derivatives from late-log cultures grown in M9 with glucose and Casamino Acids (referred to hereafter as M9-CAA). KPPR1S produces about 7 µg UA/OD_600_, whereas the Δ*manC* capsule mutant produces about 1 µg/OD_600_. The DBD mutants produced about 20 to 30% less UA than KPPR1S (5 to 6 µg/OD_600_) (Fig. 1A). This reduction is slight and did not reflect the striking differences we observed in colony morphology, so we turned to the mucoviscosity assay. HMV strains do not sediment well during centrifugation, and the supernatant remains turbid. Measurement of the turbidity after centrifugation can therefore serve as a quantitative indicator of HMV. The strains were grown as described above, diluted to an optical density at 600 nm (OD_600_) of 1, and then subjected to low-speed centrifugation. The supertnatant OD_600_ of KPPR1S was 0.3, and, consistent with the string test result, a similar value was obtained for the Δ*rmpC* mutant (Fig. 1B). The remaining mutants all formed tight pellets with nearly cleared supernatants (OD_600_ < 0.08). These results indicate that all of these DBD mutants have decreased UA content but that UA levels do not necessarily correlate with mucoviscosity.

### Mucoviscosity impacts host cell associations.

**FIG 2:**
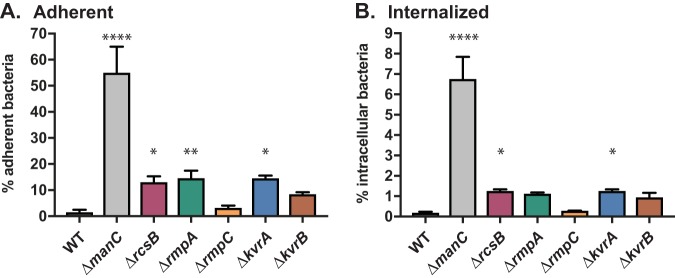
DBD mutants have altered host cell associations. BMDMs were inoculated with the indicated strains at an MOI of 50 and allowed to interact for 1 h. Adherent (A) and intracellular (B) bacteria were determined as described in Materials and Methods. The data presented here are from a representative assay. The strains used are the same strains used in Fig. 1. The one-way ANOVA test was performed to determine statistically significant differences between each mutant and WT. *, *P ≤ *0.05; **, *P ≤ *0.01; ****, *P ≤ *0.0001.

As capsule is known to have a number of protective roles during infection, we wanted to assess the phenotypes of the DBD mutants using *in vitro* models. Capsule is known to be an antiphagocytic factor and to block adherence to mammalian cells (reference 1 and references therein). Using bone marrow-derived macrophages (BMDM), we assayed the adherence and internalization of the DBD mutants. KPPR1S was barely adherent, with 1.5% of the inoculum recovered. The Δ*manC* strain was recovered at 55% of the inoculum, an ∼35-fold increase over the KPPR1S strain (Fig. 2A). The Δ*rmpC* strain exhibited a wild-type-like phenotype with very low adherence at 3%, whereas the other strains had intermediate adherence levels (8 to 15%). An identical trend in phenotypes was observed for internalized bacteria. Only 0.2% of the KPPR1S inoculum was internalized, but nearly 7% of Δ*manC* was intracellular (Fig. 2B). The DBD mutants each measured about ∼1%, with the exception of the Δ*rmpC* mutant at 0.3%. In each assay, the Δ*kvrA*, Δ*kvrB*, Δ*rmpA*, and Δ*rcsB* strains showed between 5- to 8-fold increases in host-cell associations compared to KPPR1S. All five of the DBD mutants produced similar levels of capsule, but only the Δ*rmpC* strain retained the HMV phenotype. Thus, it appears that HMV, rather than UA content, is a more critical determinant in avoiding host cell associations.

### Attenuation of the DBD mutants reveals different roles in virulence.

**FIG 3:**
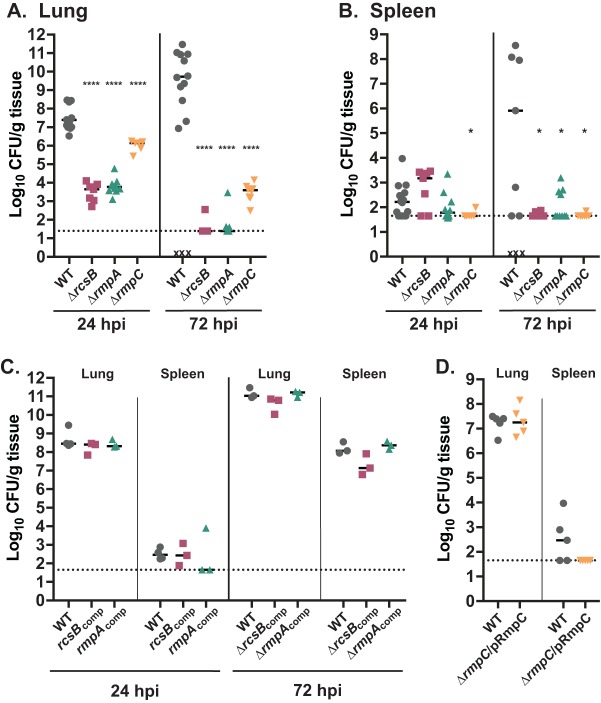
DBD mutants are attenuated in a mouse pneumonia model. Mice were inoculated with 2 × 10^4^ CFU of the indicated strains and euthanized at 24 or 72 hpi. Lungs and spleens were removed, macerated, and plated for determination of bacterial burden as CFU per gram of tissue. The data in panels A (lungs) and B (spleens) were compiled from three experiments. Representative experiments with complemented strains are presented in panels C and D. The strains used are KPPR1S (WT), VK248 (Δ*rcsB*), VK352 (Δ*rmpA*), VK487 (Δ*rmpC*), VK532 (*rcsB*_comp_), VK379 (*rmpA*_comp_), and VK487 with pKW185 (pRmpC). The Mann-Whitney test was performed to determine statistically significant differences between mutants and WT. *, *P ≤ *0.05; ****, *P ≤ *0.0001.

A mouse model of pneumonia was used to assess the *in vivo* impact of these loss-of-function mutations. C57BL/6j mice were intranasally inoculated with 2 × 10^4^ CFU and sacrificed 24 and 72 h postinoculation (hpi) to determine bacterial burdens in the lungs and spleens. Given the defects in capsule, we anticipated that each mutant would be attenuated and this was indeed observed. In the lungs at 24 hpi, the Δ*rcsB* and Δ*rmpA* strains had colonization levels about 4 logs lower than that of strain KPPR1S (Fig. 3A). The Δ*rmpC* strain was also attenuated, with nearly 2 logs less bacteria recovered from the lungs than KPPR1S. At 72 hpi, the lungs of mice inoculated with the Δ*rcsB* or Δ*rmpA* strains were nearly cleared, and the burden from those inoculated with the Δ*rmpC* mutant was very low. In the spleens at 24 hpi, the levels of strain KPPR1S are typically very low. Although not significantly different, the median burden of the Δ*rcsB* mutant was about 1,500 CFU/g, while that of KPPR1S was 165 CFU/g (Fig. 3B). Nearly all mice inoculated with the Δ*rmpC* mutant had undetectable CFU in the spleens, and this was a significant reduction compared to KPPR1S. By 72 hpi, each DBD mutant showed a significant decrease in bacterial burden in the spleens compared to KPPR1S. Although the CFU recovered from mice infected with the Δ*rcsB* mutant was higher than KPPR1S at 24 hpi, it fell to barely detected levels at 72 hpi. Chromosomal complementation of the *rmpA* and *rcsB* genes restored the bacterial burdens to wild-type levels at each time point (Fig. 3C). For reasons we do not understand, we were unable to generate the plasmid needed for chromosomal complementation of the *rmpC* gene. We therefore tested a strain carrying a plasmid-borne copy of *rmpC*, which showed complementation in the lungs at 24 hpi (Fig. 3D); no bacteria were recovered at 72 hpi, indicating that the plasmid likely had been lost during the infection (data not shown). Complementation was not observed in the spleen; this could be due to loss of the plasmid during dissemination or to potential consequences from multicopy expression.

### Gene expression at the *cps* locus is altered in DBD mutants.

**FIG 4:**
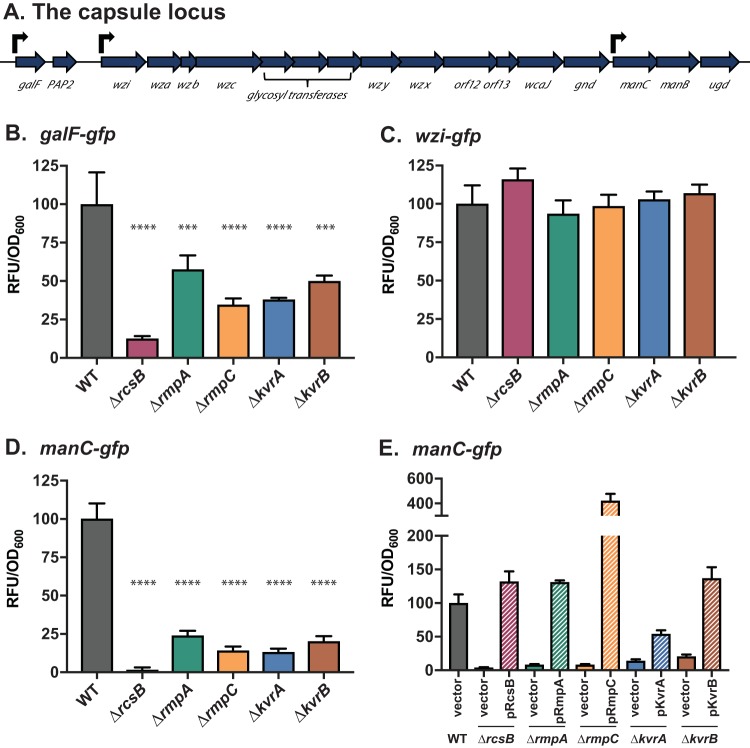
Capsule gene expression is affected by loss of DBD genes. (A) Schematic of the capsule locus containing genes for sugar precursor biosynthesis, polymerization, and export. (B to E) Saturated cultures of WT and mutant strains carrying plasmids with transcriptional *gfp* fusions were subcultured and grown as described in the legend to Fig. 1. Relative fluorescence units (RFU) were measured, normalized first to the culture OD_600_, and then to WT (set at 100). The three characterized promoters tested were *galF-gfp* (pPROBE_galF) (B), *wzi-gfp* (pPROBE_wzi) (C), *manC-gfp* (pPROBE_manC) (D). (E) Complementation assays were performed using strains transformed with *manC-gfp* and individual complementation plasmids (pRcsB [pKW173], pRmpA [pKW184], pRmpC [pKW185], pKvrA [pTM006], and pKvrB [pTM007]). The one-way ANOVA test was performed to determine statistically significant differences between each mutant and WT. ***, *P* ≤ 0.001; ****, *P ≤ *0.0001.

Because *rcsB*, *rmpA*, *rmpC*, *kvrA*, and *kvrB* are known or predicted to encode proteins with DNA binding domains and mutations in these genes affected capsule-associated phenotypes, we sought to examine whether these mutations impacted *cps* expression. The *cps* locus contains three characterized promoters, located upstream of *galF*, *wzi*, and *manC* (Fig. 4A). The regions upstream of these genes that should contain promoters were cloned into a *gfp* reporter plasmid that was then transformed into strain KPPR1S and each DBD mutant to assay expression levels. Expression from the *wzi* promoter was not altered in the mutants, suggesting that it is not regulated by any of these proteins (Fig. 4C). The *galF* and *manC* promoters were both affected by all the mutants, including the Δ*rmpC* mutant (Fig. 4B and D). Both promoters had decreased expression compared to WT, ranging from a 2- to 7-fold reduction in *galF* expression (Fig. 4B) and a 4- to 39-fold reduction in *manC* expression (Fig. 4D). Plasmids containing the individual regulator genes were transformed into the respective DBD mutants with the *manC-gfp* reporter, and restored (or enhanced) expression was observed when the gene is expressed in *trans* (Fig. 4E). Thus, the capsule alterations described above may be a consequence of reduced expression of the biosynthetic genes.

### KvrA, KvrB, and RcsB activate the *rmpA* promoter.

**FIG 5:**
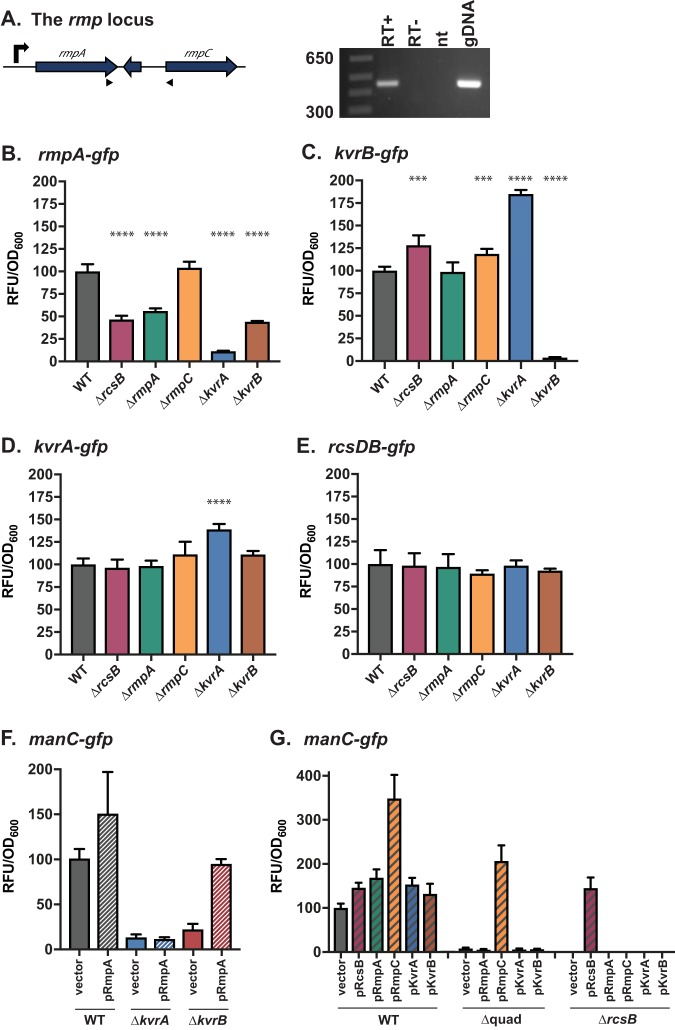
RcsB, KvrA, and KvrB control *rmpA* expression. (A) *rmpC* is in an operon with *rmpA*. Standard PCR was performed with primers CB472 and CB498 (black arrows) using wild-type genomic DNA (gDNA), samples from cDNA synthesis reactions with reverse transcriptase (RT+) and without reverse transcriptase (RT−), or with no template (nt). The RNA used to generate the cDNA was isolated from strain KPPR1S grown in LB from a previously published data set (23). Promoter-*gfp* fusions for *rmpA* (*rmpA-gfp*, pKW174) (B), *kvrB* (*kvrB-gfp*, pPROBE_kvrB) (C), *kvrA* (*kvrA-gfp*, pPROBE_kvrA) (D), and *rcsDB* (*rcsDB-gfp*, pKW170) (E) were transformed into the indicated strains and grown as described in the legend to Fig. 1. (F and G) Strains containing *manC-gfp* were transformed with the indicated complementing plasmids; plasmid names are as given in the legend to Fig. 4. The one-way ANOVA test was performed to determine statistically significant differences between each mutant and WT. ***, *P ≤ *0.001; ****, *P ≤ *0.0001.

When multiple regulators impact transcription, questions arise as to whether each individually impacts transcription or whether some act indirectly by regulating the direct regulator. To begin to address these questions, we cloned the putative promoter regions upstream of each DBD gene into our *gfp* reporter plasmid and transformed them into KPPR1S and mutant strains. We were unable to detect expression from the region upstream of *rmpC* and tested to determine whether it was in an operon with *rmpA*. Using primers that flank the intergenic region between *rmpA* and *rmpC*, a product was obtained when cDNA or genomic DNA was provided as a template, but not in the negative controls (Fig. 5A), thus indicating that *rmpC* is in an operon with *rmpA*. RmpA and KvrB autoregulate their own expression by about 2- and 10-fold, respectively (Fig. 5B and C), while expression of *kvrA* and *rcsB* was largely unregulated (Fig. 5D and E). Expression of *rmpA* was dependent on KvrA, KvrB, and RcsB; loss of KvrA resulted in a 9-fold reduction in *rmpA* levels, and loss of KvrB or RcsB resulted in 2- and 2.5-fold reductions, respectively (Fig. 5B). This prompted us to determine whether the decreased expression from the *galF* and *manC* promoters in Δ*kvrA*, Δ*kvrB*, and Δ*rcsB* strains were due to loss of expression from the *rmpA* promoter. We therefore transformed pRmpA into these strains carrying the *manC*-*gfp* reporter to determine whether an extrachromosomal copy of *rmpA* could compensate for loss of *kvrA*, *kvrB*, or *rcsB*. No changes in *manC* expression were observed in the Δ*kvrA* and Δ*rcsB* strains, but pRmpA did restore *manC* expression in the Δ*kvrB* mutant (Fig. 5F and G). These data suggest that KvrA and RcsB have an impact on the *manC* promoter independent of their impact on *rmpA* expression but that the impact of KvrB on expression may solely be indirect, through regulation of the *rmpA* promoter.

To further dissect this network, we constructed a strain lacking *kvrA*, *kvrB*, *rmpA*, and *rmpC*, referred to as Δquad. We transformed pKvrA, pKvrB, pRmpA, or pRmpC into the Δquad strain containing the *manC-gfp* reporter and assayed *manC* expression. We attempted to address the role of RcsB using a strain lacking all five regulator genes, but this strain displayed some pleotropic defects and the data were unreliable, so these same plasmids along with pRcsB were transformed into the Δ*rcsB* strain. Ectopic expression revealed that *rmpC*, but not *rmpA*, *kvrA*, or *kvrB*, restored *manC* expression in the absence of the other three regulators, and this is observed only if *rcsB* is present (Fig. 5G). Very high levels of *manC-gfp* were detected from the Δquad strain with pRmpC, but no expression was detected from Δ*rcsB* with pRmpC. Consistent with the data from the Δquad strain, neither pKvrA, pKvrB, nor pRmpA could restore expression of *manC-gfp* when transformed into the Δ*rcsB* mutant. Collectively, these data suggest that RcsB is required for any level of *manC* transcription and that plasmid-encoded RmpC (thus likely produced at higher levels) leads to high levels of *manC* expression, even in the absence of *rmpA*, *kvrA*, and *kvrB*.

### RmpA and RmpC have overlapping and independent roles.

**FIG 6:**
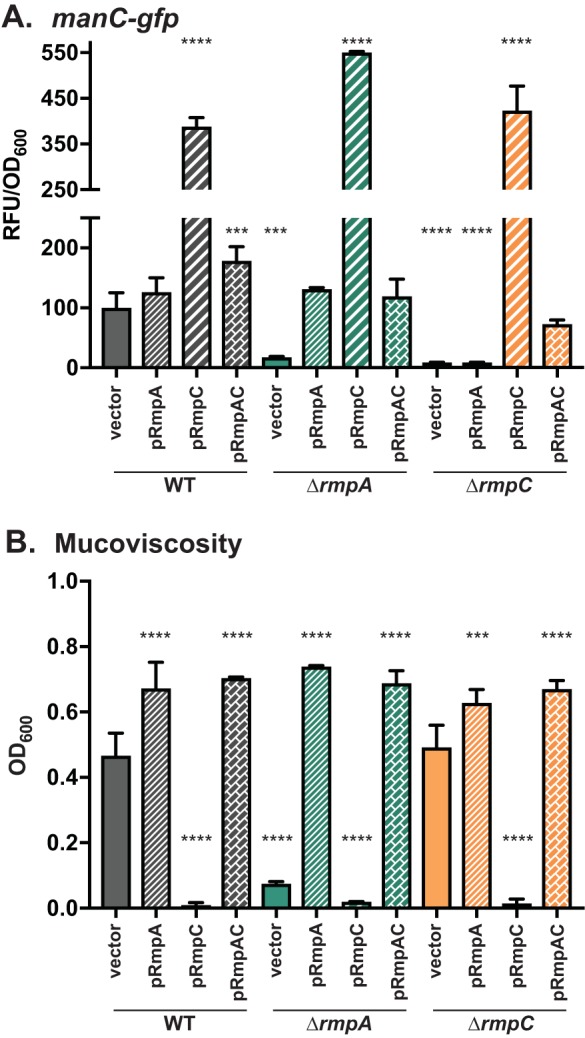
RmpA and RmpC have overlapping and independent functions. The WT, Δ*rmpA*, and Δ*rmpC* strains were transformed with *manC-gfp* (pPROBE_manC) and pRmpA (pKW184), pRmpC (pKW185), or pRmpAC (pKW186), grown as described in the legend to Fig. 1 and assayed for *manC* expression (A) or mucoviscosity (B). The one-way ANOVA test was performed to determine statistically significant differences between each mutant and WT. **, *P ≤ *0.01; ***, *P ≤ *0.001; ****, *P ≤ *0.0001.

Through the course of the experiments that included plasmids for expression of *rmpA* or *rmpC*, we noted some intriguing phenotypes. First, strains carrying pRmpC have exceptionally high expression of *manC-gfp* at levels 4- to 5-fold higher than that of the WT (Fig. 4E and 5G). Second, broth cultures of strains carrying pRmpA become viscous after several hours of growth with the inducer anhydrous tetracycline (aTc). We decided to examine this more closely by transforming KPPR1S, Δ*rmpA*, and Δ*rmpC* strains with either pRmpA, pRmpC, or pRmpAC and measuring mucoviscosity and *manC-gfp* expression. As before, introduction of pRmpC resulted in very high levels of *manC-gfp* expression in each mutant (Fig. 6A). Introduction of pRmpA only restores expression in Δ*rmpA*, indicating that RmpC is essential for maximal *manC* expression. Curiously, introduction of pRmpAC, presumably overexpressing both genes, resulted in *manC-gfp* levels similar to that of the WT in all three mutant strains. Thus, it appears that the stoichiometry of RmpA and RmpC is important for normal expression from the *manC* promoter. Although pRmpC led to high *manC-gfp* expression, it also led to a striking reduction in mucoviscosity in all strains, including WT (Fig. 6B). Strains with pRmpA showed a significant increase in mucoviscosity in all strains, consistent with its role in the HMV phenotype. Introduction of pRmpAC into the WT, Δ*rmpA*, and Δ*rmpC* strains led to increased mucoviscosity similar to that seen with these strains containing pRmpA, even though strains with pRmpAC have normal levels of *manC-gfp* expression. Collectively, these data show that RmpA is necessary for “normal” expression of *manC* and for the HMV phenotype. RmpC is necessary for full *manC* expression, and overexpression leads to elevated *manC* expression but loss of HMV. Thus, it appears that the relative amounts of RmpA and RmpC are critical for the HMV phenotype as well as *cps* expression. Importantly, these data indicate that while there is some cooperativity between RmpA and RmpC, they do not perform identical functions. Furthermore, expression levels of capsule biosynthetic genes do not necessarily correlate with HMV, hinting that the composition of the extracellular material that produces this phenotype may not be identical to the polysaccharides that comprise the capsule.

## DISCUSSION

In this work, we introduce a new capsule regulator, RmpC, and we have shown that RcsB and RmpA contribute to capsule gene expression and production of the HMV phenotype in the hypervirulent ATCC 43186 derivative KPPR1S. The roles of RcsB and RmpA were also identified as contributing to capsule production in this strain using methodology that specifically selected for mutants with altered hypermucoidy (25). The fact that RcsB and RmpA regulate capsule gene synthesis in strain KPPR1S is not surprising, as they have had this role in every other *Klebsiella* strain examined. However, new information indicates there is a regulatory cascade in place by which several regulators encoded by the K. pneumoniae core genome (KvrA, KvrB, and RcsB) control *rmpA* expression in addition to *cps* expression. RmpA in turn autoregulates its expression, and that of *rmpC* as well, since they are coexpressed and no promoter could be detected upstream of *rmpC*. Furthermore, experiments with *rmpA* and *rmpC* mutants suggest that RmpA and RmpC have overlapping and separate functions that contribute to *cps* expression and the HMV phenotype. Last, although each of these regulators is required for *cps* expression, they have different virulence phenotypes in a mouse pneumonia model, suggesting that their roles extend beyond capsule regulation.

As the polysaccharide capsule is a critical virulence factor in K. pneumoniae, its production is tightly controlled. Numerous transcriptional regulators have been identified as contributing to capsule gene expression in various K. pneumoniae strains. In addition to the Rcs phosphorelay system and RmpA, these include CRP, H-NS, IscR, the response regulators KvhA, KvgA, and KvhR, and the newly reported regulators KvrA and KvrB (10, 12, 14, 16, 17, 23). A considerable amount of work has been dedicated to the Rcs phosphorelay system, and many of the mechanisms involved in regulation of and by this system have been elucidated in E. coli (9), and identification of the RcsAB box upstream of *galF* in K. pneumoniae suggests that its function is likely conserved (8). RmpA was identified in the late 1980s as contributing to the HMV phenotype (17) and appears to be primarily limited to the *Klebsiellae*; however, its mechanism of action remains largely unknown. In the HMV+/K2 type strain GC43, RmpA was shown to interact with RcsB, and both proteins were required for activation of capsule gene expression (26). The HMV+/K1 type strain NTUH-K2044 contains both chromosome- and plasmid-encoded copies of *rmpA* that are nearly identical (>90% amino acid identity). One study reported that only the plasmid-borne gene, and not the chromosomal copy, impacted capsule production *in vitro*, but neither gene appeared to contribute to virulence in mice inoculated intragastrically or intraperitoneally (19). This contradicts results obtained from two different HMV+/K2 type strains that showed significant increases in the 50% lethal dose (LD_50_) for *rmpA* mutants compared to the isogenic WT in intraperitoneal models (17, 26). One conundrum of this body of research is that most of these regulators were studied for their independent contributions using only single deletion strains. Further complicating the interpretation is that these studies have been conducted using a variety of different K. pneumoniae strains that may or may not have the same complement of regulators, and therefore have potentially varying mechanisms of regulation of *cps* and other virulence genes.

**FIG 7:**
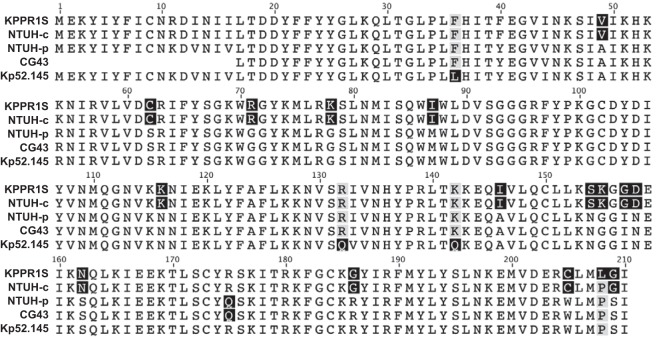
Alignment of RmpA amino acid sequences from *hv* strains. DNA sequences were obtained from the nucleotide accession numbers, translated, and aligned using Geneious v5.3.6. The strains, accession numbers, and open reading frames were as follows: KPPR1S, GenBank accession no. CP009208.1, ORF VK055_5097 (36); NTUH-c, accession no. AP006725, ORF KP1_3619 (37); NTUH-p, accession no. AP006726, ORF KP1_p020 (37); CG43, accession no. AY378100.1 (pLVPK) (20), ORF LV255; Kp52.145, accession no. (plasmid II) NZ_FO834905, ORF BN49_RS00655 (22).

The *rmpA* and *rmpC* genes in strain KPPR1S are nearly identical to those on the chromosome of NTUH-K2044 (Fig. 7), and the protein sequences show only one amino acid change in RmpA right near the C terminus and complete identity in RmpC. Comparing strain KPPR1S to the NTUH-K2044 plasmid copies, there are 17 different residues in the RmpA protein and 15 differences in RmpC. The other two strains in which RmpA has been studied each contain only one copy of *rmpA* on large virulence plasmids (17, 20, 22), and both more closely match that of the NTUH-K2044 plasmid. However, these changes must have only subtle or no effects, as all the RmpA proteins appear to be functional in some context, except perhaps the NTUH-K2044 chromosomal copy. Expression from the NTUH-K2044 *manC* promoter is reduced when expressed in the KPPR1S Δ*rmpA* mutant compared to the WT KPPR1S strain, and expression is restored to WT levels when either NTUH-K2044 or KPPR1S *rmpA* is expressed in *trans* (K. A. Walker and V. L. Miller, unpublished results). This suggests that the NTUH-K2044 chromosomal copy has the potential for functionality even though this was not evident when examined (19). It is possible that the plasmid and chromosomal promoters are differentially regulated in response to different signals, such that only the plasmid copy is produced or active in broth (where *cps* expression was tested). Infection data with a mutant lacking both copies of *rmpA* was not reported (19); thus, the lack of attenuation observed from the single mutants could be due to functional redundancy *in vivo*. Another key difficulty in interpreting the prior work with RmpA is that *rmpC* had not been identified, and thus, the impact of *rmpA* mutations on *rmpC* expression and function were not taken into account.

In strain KPPR1S, there is severe attenuation of the Δ*rmpA* strain in our pneumonia model, and this phenotype is nearly identical to the phenotypes of the Δ*rcsB* and Δ*kvrA* strains (23). Curiously, the level of attenuation observed for the Δ*rmpC* strain is not as severe, despite the similar decreases in *galF* and *manC* expression and UA levels in these mutants. The Δ*kvrB* mutant also has an intermediate phenotype, with lung colonization levels higher than those of the Δ*rmpC* strain at 72 hpi (23). Therefore, the changes in *cps* expression observed *in vitro* cannot fully account for the virulence defects in these DBD mutants. Consistent with this notion, the Δ*rmpC* strain behaves like the WT in adherence and phagocytosis assays. The capacity to avoid host cell contact and uptake appears to be driven more by HMV rather than by *cps* expression levels, and the retention of HMV by Δ*rmpC* may be why it is not as severely attenuated in the pneumonia model.

Although a regulatory cascade exists in which RcsB, KvrA, and KvrB control *rmpA* expression, RcsB and KvrA also appear to have impacts on *manC* expression beyond regulating *rmpA*. This is evidenced by the lack of complementation of *manC* expression in the Δ*rcsB*, Δ*kvrB*, or Δ*kvrA* strains when *rmpA* was expressed in *trans*. However, overexpressing *rmpC* does complement for loss of all regulators except RcsB. Because no other regulator tested could compensate for the loss of RcsB and because *manC* expression is barely (and often not at all) detected in the Δ*rcsB* mutant, it appears that RcsB is necessary for basal level expression from the *manC* promoter. One function of the other regulators may be to elevate *cps* expression above this basal level, perhaps in response to different signals.

The differences in virulence assays between the Δ*rmpA* and Δ*rmpC* mutants are particularly intriguing. That these genes are chromosomal neighbors suggests they have overlapping functions. However, the distinct mucoviscosity phenotypes of these mutants indicate that their functions are separate. The Δ*rmpA* mutant lost the HMV phenotype and is string test negative, while the Δ*rmpC* mutant is string test positive and HMV positive, yet both mutants display similar decreases in *manC* expression and UA levels. It had been speculated that the HMV phenotype is due to an overabundance of capsule (27), but the investigators that identified RmpA concluded HMV was not due to overproduction of capsule because their nonmucoid *rmpA* mutant strain produced an equivalent amount of capsule as the parent strain (17). The phenotypes of the Δ*rmpC* strain support the notion that HMV is not simply due to an overabundant capsule. When the Δ*rmpA* or Δ*rmpC* mutation is complemented in *trans*, the defects in expression and virulence are restored, but overexpression leads to some other interesting phenotypes. First, overexpression of *rmpA* leads to increased HMV such that the broth cultures become viscous. In the Δ*rmpC* strain with pRmpA, HMV increases but *manC* expression is not restored. This is a key indicator that HMV is not necessarily dependent on capsule production. Second, overexpression of *rmpC* leads to very high levels of *manC* expression (even in the absence of other regulators) but a complete loss of HMV, even in WT. Thus, it appears that RmpA is primarily responsible for the HMV phenotype and that RmpC is primarily responsible for *cps* expression. Expression of *rmpC* will be reduced in a *rmpA* mutant due to the loss of positive autoregulation by RmpA. We speculate that the association of *rmpA* as a regulator of *cps* transcription may actually be due to effects on *rmpC* expression, as most strains we examined that have the *rmpA* gene also have *rmpC*. Using tBLASTn and selecting only complete sequences (whole chromosome or plasmid), we identified about 40 strains that encode both RmpA and RmpC; from the available sequences, only three were found to encode RmpA alone. Further supporting the notion that RmpA is an indirect regulator of *cps* transcription is that overexpression of *rmpA* does not restore *manC* expression in the Δ*rmpC* strain. Despite these distinct roles, these proteins do appear to have some coordinated function. Overexpressing both genes in either the Δ*rmpA* or Δ*rmpC* mutant restores WT level of *manC* expression, although HMV is still elevated. Thus, the ratio of RmpA to RmpC seems to be an important aspect of *cps* expression. Proteins containing LuxR-type DNA binding domains are known to dimerize. Several models can be envisaged, assuming dimerization occurs. The simplest model proposes that RmpA homodimers regulate HMV and RmpC homodimers regulate *cps* expression. In this scenario, RmpA-RmpC heterodimer could serve to sequester RmpA and RmpC, thus controlling appropriate levels of both HMV and *cps* transcription by affecting the concentration of the homodimers. When the balance is shifted by overexpression of one regulator, the ratio of homodimers is skewed to favor increased HMV or *cps* expression. It is also possible that the RmpA-RmpC heterodimer binds DNA and thus directly impacts HMV and *cps* expression. RmpA was shown to interact with RcsB (26), and it is plausible that the RmpA-RcsB heterodimer regulates HMV. An abundance of RmpC could thus block HMV by preventing the formation of the RmpA-RcsB dimer through sequestration of RmpA. These models are clearly overly simplistic, as they ignore the contributions of the other regulators, but they provide a framework for future experimentation. Ongoing experiments will test these models and probe the individual regulons to identify other virulence-related genes as well as those that contribute to HMV. A greater understanding of how each regulator functions within the context of other regulators and within a single strain will provide much needed information about the production of capsule and HMV and may promote the development of new therapeutics against this problematic pathogen.

## MATERIALS AND METHODS

### Bacterial strains and growth conditions.

Table 1 lists the bacterial strains and plasmids used in this work. E. coli strains were grown in LB medium at 37°C. K. pneumoniae strains were grown in LB at 37°C for mouse and BMDM infections or M9 medium supplemented with 0.4% glucose and 0.2% Casamino Acids for capsule regulation and production assays. Where appropriate, antibiotics were added at the following concentrations: kanamycin (Kan), 50 µg/ml; rifampin (Rif), 30 µg/ml, spectinomycin (Sp), 50 µg/ml. For expression of genes cloned into pMWO-078, 50 or 100 ng/ml anhydrous tetracycline (aTc) was added to the media at the time of subculture.

**TABLE 1:** Bacterial strains and plasmids used in this work

Strain or plasmid	Relevant genotype or phenotype	Source or reference
E. coli		
DH5α	F^−^ p80Δ*lacZM15* Δ(*lacZYA*-*argF*)*U169 deoP recA1 endA1 hsdR17* (r_K_^−^ m_K_^−^)	Invitrogen
S17-1λ*pir*	Tp^r^ Str^r^ *thi pro hsdR hsdM*^+^ RP4::2-Tc::Mu::Km Tn*7* λ *pir* lysogen	38

K. pneumoniae		
KPPR1S	ATCC 43816, Rif^r^ Str^r^	39
VK248	KPPR1S, Δ*rcsB*	This work
VK532	KPPR1S, *rcsB* reconstituted	
VK277	KPPR1S, Δ*kvrA*	23
VK352	KPPR1S, Δ*rmpA*	This work
VK379	KPPR1S, *rmpA* reconstituted	This work
VK410	KPPR1S, Δ*kvrB*	23
VK429	KPPR1S, Δ*kvrA* Δ*kvrB* Δ*rmpAC* (Δquad)	This work
VK487	KPPR1S, Δ*rmpC*	This work
VK506	KPPR1S, Δ*manC*	This work

Plasmids		
pPROBE	Kan^r^; *gfp* transcriptional reporter vector	29
pKAS46	Kan^r^; MobRP4 *ori*R6K, cloning vector	28
pMWO-078	Sp^r^; p15A *ori* cloning vector, *tetO*	30
pCB096	*rmpA* in-frame deletion in pKAS46	This work
pCB109	*rmpAC* in-frame deletion in pKAS46	This work
pKW189	*rmpC* in-frame deletion in pKAS46	This work
pCB058	*rcsB* in-frame deletion in pKAS46	This work
pCB043	*manC* in-frame deletion in pKAS46	This work
pCB112	*rmpA* region in pKAS46	This work
pKW190	*rcsB* region in pKAS46	This work
pKW173	*rcsB* in pMWO-078	This work
pKW184	*rmpA* in pMWO-078	This work
pKW185	*rmpC* in pMWO-078	This work
pKW186	*rmpAC* in pMWO-078	This work
pTM006	*kvrA* in pMWO-078	This work
pTM007	*kvrB* in pMWO-078	This work
pPROBE-*manC*	*manC* promoter region in pPROBE	23
pPROBE-*galF*	*galF* promoter region in pPROBE	23
pPROBE-*wzi*	*wzi* promoter region in pPROBE	23
pPROBE-*kvrA*	*kvrA* promoter region in pPROBE	This work
pPROBE-*kvrB*	*kvrB* promoter region in pPROBE	This work
pKW174	*rmpA* promoter region in pPROBE	This work
pKW170	*rcsDB* promoter region in pPROBE	This work

### Plasmid and strain construction.

The primers used for each construct are listed in Table 2. Plasmids for making in-frame deletions were generated using pKAS46 as described previously (23) with minor modifications. Briefly, fragments of 500 to 800 bp upstream and downstream of the targeted gene were amplified, digested, ligated into pKAS46, and electroporated into E. coli S17-1 λ *pir*. The resulting plasmids (pCB43, pCB058, pCB096, pCB109, and pKW189) were verified by sequencing and introduced into K. pneumoniae via conjugation. Transconjugants were selected by growing on LB agar containing Rif (30 µg/ml) and Kan (50 µg/ml). Several Rif^r^/Kan^r^ colonies were streaked onto LB agar with 2.5 mg/ml streptomycin (Str_2500_) to select for clones that had undergone the second recombination step (28). Following another isolation streak on LB-Str_2500_, Str^r^/Kan^s^ colonies were subjected to PCR to determine whether the second recombination event yielded the wild-type or mutant genotype. Strains with the resulting in-frame deletions in *rcsB*, *rmpA*, *rmpC*, and *manC* were named VK248, VK352, VK487, and VK506, respectively. Strain VK429 (Δquad) was generated by sequential deletion of *kvrA*, *kvrB*, then *rmpAC*.

**TABLE 2:** Primers used in this work

Primer	Sequence (5′→3′)^*a*^	Use^*b*^
CB184	GATCATACTAGTCAGCTA CCTGATCGACATTAC	F pCB043 5′ flank
CB185	GATCATGTGCACCAGCCATAATCACAGGAAGC	R pCB043 5′ flank
CB186	GATCATGTGCACGTCGTTGCTAATTTTTTCGGG	F pCB043 3′ flank
CB187	GATCATCAATTGGTCCACCGTGTTCCACGTC	R pCB043 3′ flank
CB326	CATGCTAGCTATAGTTCTAGAGACCCAGAGCGACCTGTAC	F pCB058 3′ flank
CB327	CTGCAGGCGGCCGCCATATGCTCACCGGTGAGCAGAGAC	F pCB058 5′ flank
CB330	GAACGTAATTATTGCCGATGACGACAAAGAGTAATCTCTTCGCCCTC	R pCB058 5′ flank
CB331	GAGGGCGAAGAGATTACTCTTTGTCGTCATCGGCAATAATTACGTTC	R pCB058 3′ flank
CB470	GATCATGATGCGGCCGCCCTTTGTTGAACAATTCCATG	F pCB096, pCB109 5′ flank, pCB112
CB471	GATCATCTGCAGCCAGTTAACTGCTTTAGACC	R pCB096 5′ flank
CB472	GATCATCTGCAGGGTTGATGAAAGATGTCTCATG	F pCB096 3′ flank
CB473	GATCATACGCGTGTCAATGATGTTAATTCCGATG	R pCB096 3′ flank, pCB112
CB474	GATCATGTCGACGATTGATATTGATGGATCAAAG	F pKW184, pKW186
CB475	GATCATGGATCCATAAATGAAAGAGTGCTTTCAC	R pKW184
CB495	GATCATGTCGACAATGTATCTCCAGCAAATGAG	F pKW185
CB496	GATCATGGATCCGGACACCAAAAGTTATACCATC	R pKW185, pKW186
CB497	GATCATGATGCGGCCGCGGTTTTACCCTAAAGGGTGTG	F pKW189 5′ flank
CB498	GATCATGTCGACCACAATCTCATTATTTTTCTTACC	R pKW189 5′ flank
CB499	GATCATGTCGACCCACTTAGCTTATTAAAGATGG	F pCB109, pKW189 3′ flank
CB500	GATCATACGCGTCAAAACAGCCTAAATACATTGTTG	R pCB109, pKW189 3′ flank
CB501	GATCATGTCGACCCAGTTAACTGCTTTAGACC	R pCB109 5′ flank
KW308	GCGTCGACTTGCTGCTCTGGCGCAAACCGC	F pKW173
KW309	ATAAGAATGCGGCCGCCGCTGTCTGGAATCGGGGATGG	R pKW173
KW321	ATAAGAATGCGGCCGCGAGCGTCCTGCATCACACCGGACG	F pKW190
KW322	ATCGACGCGTATAACGACGACATGATGATCCTCG	R pKW190
KW334	GCGTCGACAATCAGTATAATAACTTAGCAAGC	F pTM006
KW335	CGGGATCCAATTAATCTTTAGCCTGGAGATCG	R pTM006
KW336	GCGTCGACGTCACAATAGTTATTATATCGGCTG	F pTM007
KW337	CGGGATCCTTAGCGTAGAGAGTCCGCTAGCAC	R pTM007
MP245	CAGTACGAATTCCTAAACGTGCCAGATCAGAAC	R pPROBE_kvrA
MP246	TGCATATCTAGAAAGACTCAGTTTCACAAGCAA	F pPROBE_kvrA
MP334	TGCATATCTAGACTCTTTCGCTATCTTCCTCTG	F pPROBE_kvrB
MP335	CAGTACGAATTCTCATGGCGGCTGGCGCGG	R pPROBE_kvrB
MP353	CAGTACGAATTCACCATCAAAATGAAGAAGCGG	F pKW170
MP354	TGCATATCTAGAAAGATGTTTCAAAGTTCCCAC	R pKW170
MP357	CAGTACGAATTCCAGTTAACTGCTTTAGAC	F pKW174
MP358	TGCATATCTAGAGTCGGAGCAATCGCCAAATAC	F pKW174

a Restriction enzyme sites are underlined.

b F, forward primer; R, reverse primer.

Plasmids for *gfp* transcriptional fusions were generated by amplifying a 500- to 700-bp region upstream of the predicted start codon of the target gene, digesting the product, ligating it into pPROBE-tagless (29), and electroporating into E. coli DH5α. The resulting plasmids, pKW170 (*rcsDB*-*gfp*) and pKW174 (*rmpA-gfp*), were verified by sequencing and then introduced into K. pneumoniae by electroporation.

Plasmids for complementation in *trans* were similarly constructed; PCR-amplified inserts were ligated into pMWO-078 (30). These plasmids, pKW173 (pRcsB), pKW184 (pRmpA), pKW185 (pRmpC), pTM006 (pKvrA), and pTM007 (pKvrB), were verified by sequencing and then introduced into K. pneumoniae by electroporation.

Chromosomal complementation was done by allelic exchange. The plasmids for chromosomal complementation were generated by amplifying a single fragment that spanned the same upstream and downstream regions as the deletion constructs using the outermost primers. This insert was cloned into pKAS46 and verified by sequencing. These plasmids, pKW190 (*rcsB*) and pCB112 (*rmpA*), were introduced into the desired K. pneumoniae strain via conjugation, and the same protocol was followed as for generating in-frame deletions.

### Electroporation of plasmids into *Klebsiella*.

For efficient electroporation of plasmids into *Klebsiella*, saturated overnight cultures grown in LB at 26°C (to minimize capsule production) were subcultured into fresh LB containing 0.7 mM EDTA and grown for 2 h at 37°C (31). One milliliter of culture was washed twice with 10% glycerol, and the pellet was resuspended in 100 µl of 10% glycerol. Plasmid DNA was added to 50 µl cell suspension and subjected to electroporation. Cells were allowed to recover for 1 h in SOC medium prior to plating on LB agar with appropriate antibiotics.

### Uronic acid measurement.

The uronic acid (UA) content was measured using a modified protocol (32, 33) essentially as described previously (34). UA was extracted from a 500-ml culture with zwittergent, precipitated with ethanol, and resuspended in tetraborate/sulfuric acid. Following addition of phenylphenol, UA was detected by absorbance at 520 nm. A standard curve was generated with glucuronic acid.

### Mucoviscosity assay.

Mucoviscosity of the capsule can be assessed by low-speed centrifugation of liquid cultures (35). Various strains of K. pneumoniae were grown as in the UA assay. After the 6-h incubation, cultures were normalized to 1 OD_600_/ml and centrifuged at 1,000 × *g* for 5 min. The OD_600_ values of the supernatant were determined and plotted. HMV strains do not form tight pellets, and the supernatants therefore have higher absorbance readings.

### Transcriptional *gfp* reporter assays.

Plasmids containing various promoter-*gfp* fusions were transformed into the desired K. pneumoniae strains. The resulting strains were grown overnight in M9-CAA, subcultured in fresh medium, and grown for 6 h. Relative fluorescence units (RFU) were measured from bacterial cultures diluted 1:10 using a Synergy H1 plate reader (Bio-Tek, Winooski, WI). The OD_600_ of each culture was measured to calculate RFU/OD_600_ and then normalized to the activity from the WT strain in each assay.

### Host cell attachment and internalization assays.

BMDM were harvested from the femurs of C57BL/6j mice as described previously (23), then seeded at 5 × 10^5^cells per well in a 24-well plate, and incubated overnight in DMEM with 10% FBS. BMDM were inoculated with bacteria that were grown overnight at 37°C in LB with Rif (30 µg/ml) at a multiplicity of infection (MOI) of 50 and allowed to incubate for 1 h. For adherence assays, the BMDM medium was replaced with fresh medium containing 2 µM cytochalasin D (Sigma-Aldrich, St. Louis, MO) to halt internalization of bacteria 1 h prior to inoculation. Following incubation with bacteria, the cells were gently washed three times with 1× PBS, lysed with 0.5% saponin, diluted, and plated for bacterial CFU enumeration. For internalization assays, after a 1-h incubation with bacteria, the cells were rinsed three times with 1× PBS and then incubated in fresh medium containing 200 µM gentamicin to kill extracellular bacteria. After 30 min in gentamicin, the cells were rinsed, lysed, diluted, and plated as described above.

### Murine pneumonia model.

All animal studies were approved by the Institutional Animal Care and Use Committee of UNC-CH (protocols 14-110 and 17-033). Prior to and following inoculation, mice had unlimited access to food and water. Inoculated mice were monitored daily, and mice were euthanized upon showing signs of morbidity. Five- to 8-week-old female C57BL/6j mice (Jackson Laboratories, Bar Harbor, ME) were anesthetized by intraperitoneal (i.p.) injection with ketamine/xylazine and inoculated with 2 × 10^4^ CFU as described previously (23, 34). At the indicated time points, mice were euthanized by i.p. injection with sodium pentobarbital. Lungs and spleens were removed, macerated in 1× PBS, serially diluted, and plated for bacterial enumeration. Organ weights were recorded, and the data are presented as CFU/gram tissue.
